# Step-brothers in arms, SAGA and ATAC co-activator complexes, use different strategies

**DOI:** 10.1038/s44318-026-00892-z

**Published:** 2026-08-11

**Authors:** Francesca Rizzo, Adam Ben-Shem, H T Marc Timmers, Till Bartke, Stéphane D Vincent, Laszlo Tora

**Affiliations:** 1 Institut de Génétique et de Biologie Moléculaire et Cellulaire, Illkirch, France; 2 Centre National de la Recherche Scientifique (CNRS), UMR7104, Illkirch, France; 3 Institut National de la Santé et de la Recherche Médicale (INSERM), U1258, Illkirch, France; 4 Université de Strasbourg, Illkirch, France; 5 German Cancer Consortium (DKTK), a partnership between the DKFZ and Medical Center-University of Freiburg, Freiburg, Germany; 6 Department of Urology, Medical Center-University of Freiburg, Freiburg, Germany; 7 Institute of Functional Epigenetics (IFE), Helmholtz Munich, Neuherberg, Germany

**Keywords:** Chromatin, Transcription & Genomics

## Abstract

Transcriptional co-activator complexes play essential roles in regulating gene transcription by integrating signals from transcription factors and chromatin. Among these, the SAGA (Spt-Ada-Gcn5 acetyltransferase) and ATAC (Ada Two-A Containing) complexes represent related histone acetyltransferase assemblies that contain both shared and specific subunits. SAGA and ATAC possess similar and divergent enzymatic activities that shape their contributions to transcriptional control. Here, we review emerging evidence that highlights differential interactions of transcriptional activators with SAGA or ATAC and their differential chromatin-reading capabilities through specific recognition of histone modifications. Distinct requirements for SAGA and for ATAC have been observed in gene regulation important for maintaining cellular homeostasis and for oncogenic transformation, indicating that dysregulation of these complexes can contribute to cancer progression. The development of small-molecule inhibitors targeting key components of SAGA and/or ATAC offers promising strategies to disrupt their recruitment and transcriptional activity in cancer cells. Our review dissects the unique and overlapping functions of SAGA and ATAC, which are crucial for understanding regulation by these complexes in order to exploit them as targets in precision oncology.

## Abstract (web summary)

This review summarizes current understanding of how the SAGA and ATAC co-activator complexes regulate transcription through complementary molecular mechanisms and discusses opportunities for their therapeutic targeting in cancer.

## Graphical abstract

**Figure:**
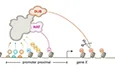

## Introduction

**Figure 1:**
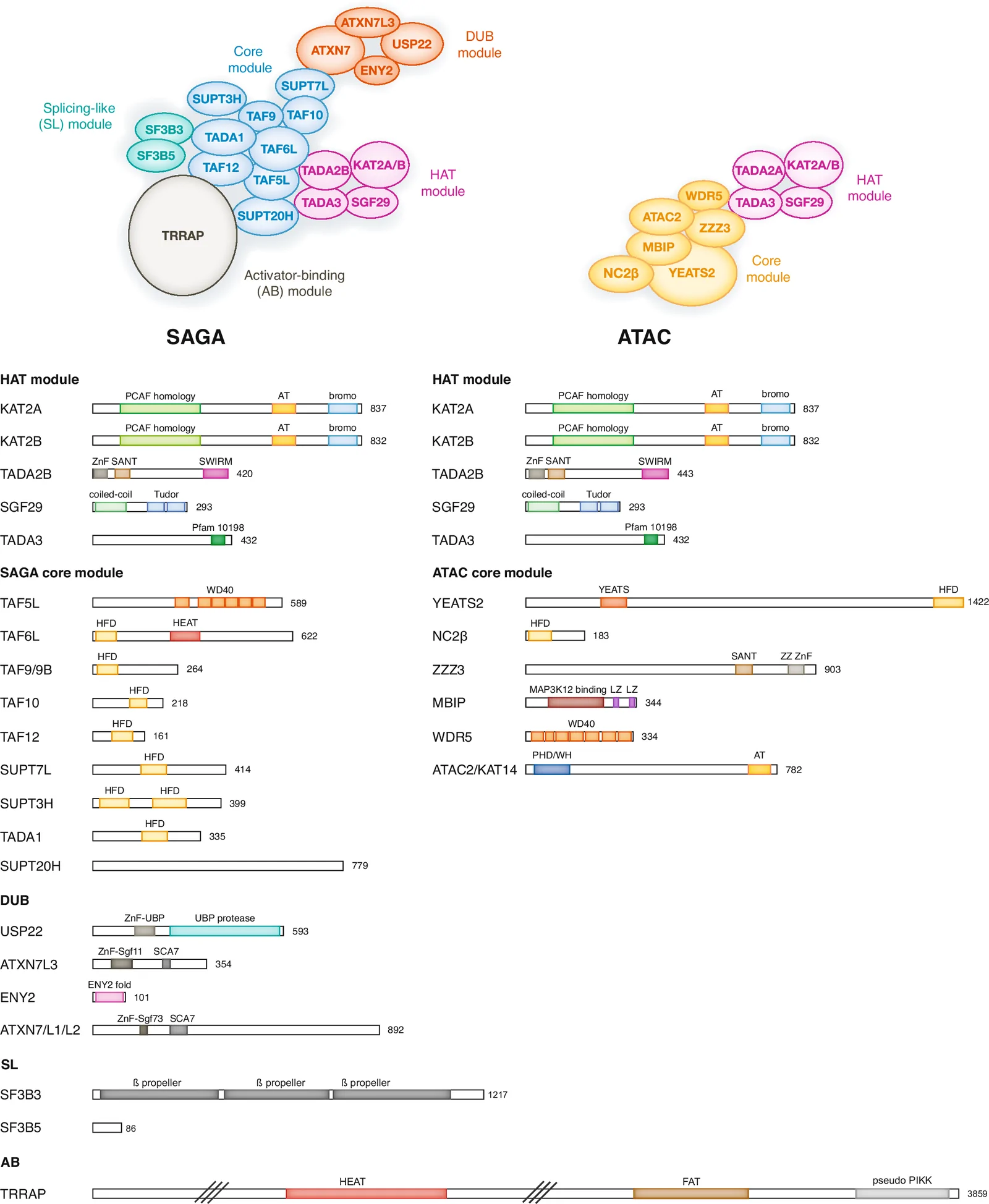
Schematic representation of SAGA and ATAC complexes. On top, the two human complexes are depicted schematically with their known functional modules represented. Below these schematic representations, each subunit belonging to either human SAGA or human ATAC complexes is represented, and their known protein domains are shown with their corresponding names. Adapted from (Helmlinger et al, 2021; Helmlinger and Tora, 2017; Spedale et al, 2012). See Table 1 for references to the different domains. Several labs identified only the ten depicted human ATAC subunits (Damilot et al, 2026; Nagy et al, 2010; Vermeulen et al, 2010; Yayli et al, 2023); however, the Host Cell factor (Hcf in *Drosophila* and HCFC1 in humans) and human MAP3K7 have also been described as ATAC complex-associated proteins from either *Drosophila* or human cell purifications (Suganuma et al, 2008; Wang et al, 2008).

Chromatin-modifying co-activator complexes are known to recognize (read), deposit (write), and remove (erase) post-translational modifications (PTMs) of histones in a dynamic manner to participate in gene transcription. Two related large multi-domain-containing co-activator complexes with histone acetyltransferase (HAT) activity, SAGA (Spt-Ada-Gcn5-acetyltransferase) and ATAC (ADA-Two-A-Containing), have been described to modify chromatin around gene promoters and to exert transcription regulatory functions (Chen and Dent, 2021; Grant et al, 2021; Helmlinger and Tora, 2017; Riss et al, 2015; Soffers and Workman, 2020; Spedale et al, 2012; Suganuma et al, 2008). Multiple lines of biochemical and genetic evidence indicate that these co-activator complexes harbor both similar and distinct activities, which differentially regulate gene expression (Fischer et al, 2021; Nagy et al, 2010; Spedale et al, 2012). The metazoan SAGA and ATAC co-activator complexes contain either 18–20 or 10 well-characterized subunits, respectively, which are organized into functional modules closely matching their arrangement into discrete structural entities (Helmlinger et al, 2021; Helmlinger and Tora, 2017). The protein composition of each module of the human SAGA or ATAC complexes and the different domains of the subunits composing their respective modules are depicted in Fig. 1 and Table 1. A given activity is carried out by one specific module, which is typically composed of several subunits containing defined protein domains (Table 1). SAGA is conserved across all three eukaryotic kingdoms and organized into five functional modules in metazoans: a) HAT, b) histone H2Bub1 deubiquitinase (DUB), c) activator binding (AB), d) splicing-like (SL) and e) core modules (Chen and Dent, 2021; Helmlinger and Tora, 2017; Herbst et al, 2021; Soffers and Workman, 2020; Spedale et al, 2012) (Fig. 1 and Table 1). Recent structural studies using cryo-EM have revealed the organization of the core+AB modules, the core+AB + HAT (three of the four subunits) module, or the core+AB + SL modules of the yeast and human SAGA complexes at high resolution (Herbst et al, 2021; Mattoo et al, 2025; Papai et al, 2020; Wang et al, 2020; Damilot et al, 2026). Interestingly, the HAT module of SAGA could only be resolved using a thermophilic fungus species, and none of the other (yeast and human) SAGA cryo-EM structures elucidated the structure of the DUB module as part of the endogenous SAGA complex. This may indicate that the HAT and the DUB modules have multiple degrees of flexibility relative to the SAGA core complex. The metazoan-specific ATAC complex is composed of two modules: the HAT and the core modules (Yayli et al, 2023). However, the structural organization of the entire ATAC complex is not yet known in molecular detail.

**Table 1:** Composition of the human SAGA and ATAC complexes and the known domain(s) of each subunit.

Modules	SAGA specific	Common	ATAC specific	Known domain(s)	Ref.
**AB**	TRRAP			Circular HEAT repeat cradle, FAT and pseudo-(Ψ)PIKK	Herbst et al, 2021
**SAGA core**	TAF5L			6xWD40 propeller	Herbst et al, 2021
	TAF6L			HFD and HEAT	Herbst et al, 2021; Scheer et al, 2012
	TAF9/TAF9B			HFD	Frontini et al, 2005
	TAF10			HFD	Trowitzsch et al, 2015
	TAF12			HFD	Birck et al, 1998
	SUPT3H			2xHFD	Birck et al, 1998
	SUPT7L			HFD	Soutoglou et al, 2005
	TADA1			HFD	Gangloff et al, 2000
	SUPT20H			–	
**DUB**	ATXN7/ATXN7L1/ATXN7L2			ZnF-Sgf73 and SCA7	Bonnet et al, 2010
	ATXN7L3			ZnF-Sgf11 and SCA7	Bonnet et al, 2010
	USP22			ZnF-UBP and DUB protease	Bonnet et al, 2008
	ENY2			ENY2 fold	Jani et al, 2012
**SL**	SF3B3			Three tandem β-propeller	Cretu et al, 2016; Sun, 2020
	SF3B5			–	
**HAT**	TADA2B			ZnF, SANT, and SWIRM	Gamper et al, 2009
		KAT2A/KAT2B (GCN5/pCAF)		Atypical E3 ligase, Bromo and AT	Nagy and Tora, 2007; Toma-Fukai et al, 2020
		TADA3		Pfam10198	Mistry et al, 2021
		SGF29		2xTudor	Bian et al, 2011
			TADA2A	ZnF, SANT, and SWIRM	Gamper et al, 2009
**ATAC core**			YEATS2	YEATS	Mi et al, 2017; Zhao et al, 2016
			ZZZ3	Myb-type HTH/SANT, ZnF-ZZ	Mi et al, 2018
			ATAC2 (KAT14 or CSR2B)	PHD/WH and AT	Callebaut and Mornon, 2012; Helmlinger et al, 2021
			MBIP	MAP3K12 inhibitory and Leucine zipper-like	Helmlinger et al, 2021
			WDR5	7xWD40 propeller	Schuetz et al, 2006; Spedale et al, 2012
			NC2b	HFD	Wang et al, 2008

SAGA and ATAC are categorized as related co-activator complexes because they share three of their four HAT module subunits, namely the enzymatic subunits KAT2A/KAT2B (GCN5/PCAF), TADA3, and SGF29 (Table 1). The fourth and distinctive subunit is either TADA2A in ATAC, or TADA2B in SAGA HAT modules, respectively (Table 1) (Gamper et al, 2009; Kusch et al, 2003; Muratoglu et al, 2003). In this respect, it is important to note that in *Drosophila* either the HAT or the DUB modules of SAGA have been isolated without the core SAGA complex, respectively, and were shown to bind chromatin independently of the core SAGA complex (Li et al, 2017; Soffers et al, 2019).

How these two co-activator complexes, harboring similar HAT modules but differing in specific subunits, regulate gene expression is not yet well understood. Here, we review recent advances describing the differential role of SAGA and ATAC in cellular homeostasis, development, and during cancerous transformation.

## Differential interactions of transcriptional activators with SAGA and ATAC complexes

An important feature of the SAGA and ATAC HAT complexes is their recruitment to the control regions of their specific target genes. It has been suggested that this targeting is mediated through DNA-specific transcription activators. Numerous studies both in yeast and in human cells have reported direct interactions between the SAGA complex and DNA-binding transcription factors (TFs) (Bhaumik et al, 2004; Brown et al, 2001; Gamper et al, 2009; Hardy et al, 2002; Ikeda et al, 1999; Jeon et al, 2025; Knutson and Hahn, 2011; Kuo et al, 2000; Liu et al, 2008; McMahon et al, 2000; Uesugi et al, 1997; Utley et al, 1998; Zhang et al, 2014). Many of these studies identified the yeast SAGA subunit Tra1, or its mammalian homolog TRRAP, as the main activator-interacting subunit of SAGA. In agreement with the potential role of TRRAP in activator interactions and shown by CUT&RUN in human HCT116 cells, the large majority of transcriptionally active genes (83%) were found to be bound by TRRAP, as expected for a transcriptional co-activator (Detilleux et al, 2022). Based on the observations described above Tra1/TRRAP has been designated as an activator binding (AB) module of SAGA (Table 1) (Elias-Villalobos et al, 2019). However, loss of the Tra1 subunit, which is expected to mediate SAGA interactions with multiple activators in cells, has only limited phenotypic and transcriptional effects in *S. pombe* (Helmlinger et al, 2011). It has also been described that human TRRAP directly represses the transcription of *IRF9*, which encodes a master regulator of interferon-stimulated genes, but whether this negative role of TRRAP in interferon gene regulation could be carried as a subunit of the human SAGA complex has not yet been demonstrated (Detilleux et al, 2022). Moreover, the fact that in *S. cerevisiae* Tra1 and in metazoan cells TRRAP is also a subunit of the NuA4/TIP60 HAT-containing co-activator complex, makes the direct activator-Tra1/TRRAP interactions within SAGA potentially less specific, as both SAGA and NuA4 would be recruited. Thus, SAGA co-activator recruitment may also rely on other subunits, which could directly interact with DNA-sequence-specific TFs. In agreement with this suggestion, other mammalian SAGA subunits, such as KAT2A, KAT2B, TADA2B, TADA3, TAF9, TAF10 or TAF12, can engage via direct physical interactions with activators (Buschmann et al, 2001; Chen et al, 2024; Chesnutt et al, 2024; Jabbur et al, 2002; Jacq et al, 1994; Liu et al, 2008; Lu and Levine, 1995; Papadopoulos et al, 2015; Pelka et al, 2009; Reeves and Hahn, 2005; vom Baur et al, 1998; Xie et al, 2014; Xu et al, 2018). In the above studies, the main activators or activation domains that were shown to directly interact with SAGA were the activation domain of the herpes simplex virus VP16, the yeast GCN4 activator protein, human P53, MYC, MYB, and MAF oncoproteins, certain nuclear receptors, the human adenovirus E1A protein, mouse GATA1, and the *Drosophila* NOTCH receptor intracellular domain (NICD) (Table 2). These and additional studies have led to a model in which DNA-sequence-specific TFs recruit SAGA to promoters to enhance nucleosome acetylation, and thus increase promoter accessibility to allow transcription initiation by RNA polymerase II (Pol II) (Fischer et al, 2019). Although how exactly SAGA contributes to transcriptional regulation has been the subject of much debate, it has been suggested that yeast SAGA is required for the transcription of all Pol II-transcribed genes. Nevertheless, the SAGA dependence of transcribed genes in yeast seems to range from weak to strong (Baptista et al, 2017; Bonnet et al, 2014b). Alternatively, it has been suggested that yeast SAGA functions mostly at inducible genes, and indirectly contributes to some extent to the transcription of all genes by regulating inducible genes that subsequently have global effects on transcription (Huisinga and Pugh, 2004; Mittal et al, 2022). In mouse embryonic stem cells (mESCs) it has been found that SAGA would function as a co-activator for several key pluripotency DNA-binding TFs (OCT4, SOX2, SALL4 and MYC) and thereby regulate the pluripotency network (Fischer et al, 2021; Seruggia et al, 2019).

**Table 2:** SAGA and ATAC complexes interact directly with distinct transcription factors.

The Table summarizes activator interactions with either SAGA or ATAC, as described in the text. Human TFs are represented in black, oncogenic fusion proteins in blue, yeast (*Saccharomyces cerevisiae*, Sc) TFs in green, *Drosophila melanogaster* (Dm) TFs in red, and viral TFs in purple. ^§^ indicates that the human ER/RXR–human SAGA/ATAC interaction has been shown with mouse TADA3, a common subunit of SAGA/ATAC (vom Baur et al, 1998). * indicates that the HAT module of human SAGA and ATAC was described to interact with VP16 presumably through human KAT2A, a common subunit of SAGA/ATAC (Chesnutt et al, 2024). Numerous interactions have been identified between various TFs and TAF subunits (i.e., TAF9, TAF10, and TAF12) that are common between human SAGA and TFIID.

As the metazoan-specific ATAC complex was discovered about 10 years after the yeast and human SAGA complexes, there is less data available about its interactions with transcriptional activators [for reviews see (Helmlinger and Tora, 2017; Spedale et al, 2012)]. The first evidence of ATAC directly interacting with TFs came from studies in *Drosophila melanogaster*. Affinity purification of the ATAC complex revealed that the *Drosophila* homolog of c-JUN, Jra (Jun-related antigen), interacts with the ATAC complex (Table 2) and also with other key components of the MAP kinase (MAPK) signaling pathway (Suganuma et al, 2010).

A recent study systemically characterized the protein-protein interaction (PPI) network of a selected set of 109 human TFs using affinity purification-mass spectrometry (AP-MS) and proximity-dependent biotinylation (BioID) mass spectrometry (MS) in human cells (Goos et al, 2022). This large PPI interactome network of TFs allowed the identification of a series of DNA-binding TFs interacting either with SAGA or with ATAC subunits. Using BioID, KLF6, MYC, and MYOD TF baits interacted with SAGA complex subunits, while E2F1, ELF1, HNF4A, KLF3, KLF12, MYC, PPARG, and TYY1 TF baits interacted with ATAC subunits (Table 2). While the detected PPIs seem to be either SAGA or ATAC complex-specific, only the MYC oncoprotein interacted with both complexes, stressing that the two co-activator complexes are involved in the function of distinct gene regulatory networks. It is also important to note that the large majority of tested DNA-sequence-specific TFs did not interact with either of the two complexes in this BioID-MS survey. Although it was suggested that the BioID method is efficient for studying transient PPIs (Varnaite and MacNeill, 2016), there may be several reasons why the majority of the TFs did not interact with either SAGA and/or ATAC. Either the non-interacting TFs use different co-activators than SAGA and/or ATAC for regulating their target genes, or the PPIs between the non-identified TFs and SAGA or ATAC are too transient to be detected by BioID MS. The second scenario would suggest that the SAGA-, or ATAC-binding TFs interact more stably with either of the two complexes, when compared to those DNA-sequence-specific TFs, which did not show SAGA or ATAC interactions by BioID.

Further studies also identified activator-ATAC subunit interactions in a BioID-MS study carried out in human cells expressing the oncogenic TAZ-CAMTA1 or YAP-TFE3 fusion proteins. These chimeric TFs resulting from chromosomal translocations are known as oncogenic drivers of epithelioid haemangioendothelioma. The study showed that the DNA-binding TFs TFE3, TAZ-CAMTA1, and YAP-TFE3 interacted with the ATAC complex (Table 2), through its YEATS2, ZZZ3, and ATAC2/KAT14 subunits (Merritt et al, 2021). These and additional experiments further suggested that the ATAC complex interacts with the C-terminal domains of TAZ-CAMTA1 and YAP-TFE3 via the ATAC2/KAT14 subunit of ATAC to modulate the Hippo-signaling pathway, of which these DNA-binding TFs are integral components.

The paired-like homeobox TF, LEUTX, is a DNA-specific enhancer binding protein and a transcriptional activator for human preimplantation. Using BioID-MS to find LEUTX interacting proteins in human cells, interactions with several ATAC-specific subunits (Table 2), such as ZZZ3, ATAC2/KAT14/CSR2B, and TADA2A, were detected (Gawriyski et al, 2023). These observations further suggest that similarly to SAGA, the ATAC complex, is involved in linking DNA-sequence specific TFs, nucleosome acetylation, promoter accessibility, and Pol II transcription. In agreement with BioID-based PPIs (Goos et al, 2022), MYC and the E2F family member E2F4 were identified as DNA-binding TFs bound to ATAC-regulated (both via YEATS2 and ZZZ3) genes in mESCs, suggesting that they recruit ATAC to regulate the transcription of this set of genes (Fischer et al, 2021).

Taken together, several observations indicate that at least partly the specific stimulatory functions of SAGA and of ATAC would be defined by the specific DNA-sequence-specific TFs. In this respect, these TFs could be categorized in general as follows: no interactions detected with either of the two complexes, specific regulatory interaction with only SAGA, specific regulatory interaction with only ATAC, interactions with both SAGA and ATAC complexes. Finally, as *cis*-regulatory elements harbor multiple TF consensus motifs acting additively or synergistically to regulate gene expression, such combinatorial output could dictate the magnitude and residency time of SAGA and ATAC recruitment to promoters and enhancers (see also below).

## Differential chromatin “reading” of SAGA and ATAC complexes

Post-translational modifications of histones and DNA methylation play crucial roles in binding chromatin and transcription regulatory factors that, by “reading” these modifications, serve to alter the structural and functional properties of the underlying chromatin (Ruthenburg et al, 2007). Like most chromatin regulators, SAGA and ATAC also contain several chromatin reader domains (see Fig. 1 and Table 1). Surprisingly, subunits constituting the core, AB, and SL modules of SAGA do not harbor typical histone binding domains, while in the subunits of the SAGA DUB and HAT modules, numerous zinc finger (ZnF) motif-containing domains and other chromatin readers such as the Bromo, Tudor, SANT, and SWIRM domains can be identified (Fig. 1 and Table 1). Interestingly, the three common subunits of the HAT modules of both SAGA and ATAC contain histone binding domains [i.e., one bromodomain (BD) in KAT2A/KAT2B, and two Tudor domains in SGF29], suggesting that the two complexes may bind to similarly modified chromatin regions through these common subunits. At their C-termini, both KAT2A and KAT2B contain highly homologous BDs (Fig. 1 and Table 1), and it has been suggested that the BD of yeast KAT2A can interact with acetyl lysines (Li and Shogren-Knaak, 2009; Nagy and Tora, 2007). SGF29 contains a tandem Tudor domain (Fig. 1 and Table 1), which was shown to bind/read H3K4me2/3 modified chromatin (Bian et al, 2011). However, the presence of distinct SANT and SWIRM chromatin recognition domains in the fourth HAT subunit of either SAGA (TADA2B) or ATAC (TADA2A) suggests differential chromatin binding properties of the two complexes. Moreover, a series of additional chromatin binding motifs are present in the ATAC core module (i.e., YEATS, MYB-type helix-turn-helix (HTH), Znf ZZ, SANT, PHD-like+wing helix (WH), Table 1) and absent in SAGA, and could differentiate the chromatin recognition pattern by the endogenous SAGA from that of the ATAC complexes.

**Figure 2:**
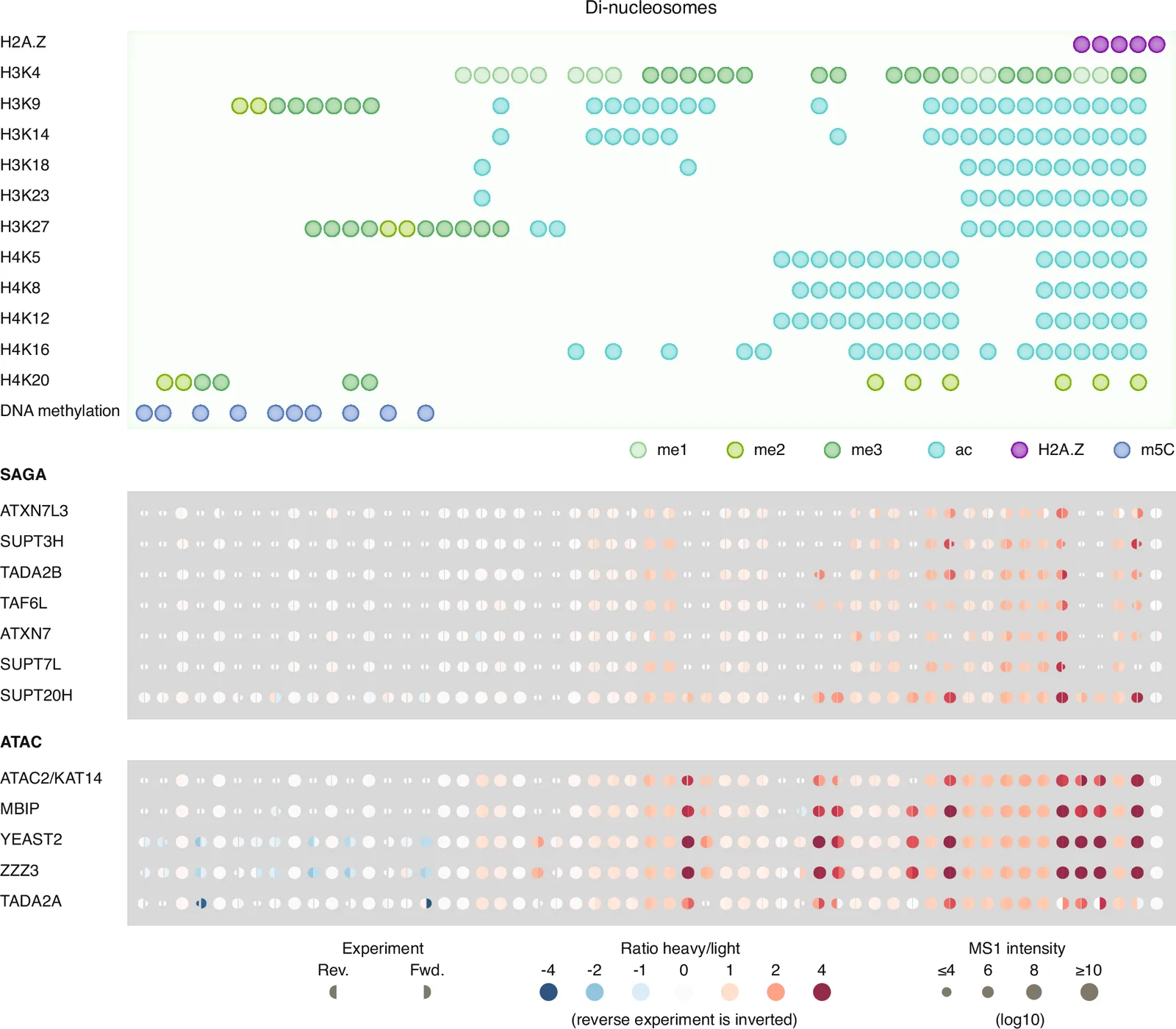
SAGA and ATAC complexes bind to various different chromatin modifications. Binding of nuclear proteins from human HeLaS3 cells to 55 differentially modified dinucleosomes was profiled by SILAC nucleosome affinity purifications (SNAP) (Lukauskas et al, 2024). The header (top panel) specifies the modification status of each dinucleosome used for pull downs. Nucleosomes are arranged in columns ordered by increasingly active chromatin state, with the respective modifications indicated in rows. Post-translational modifications (PTMs) of specific lysine residues in histone H3 and H4 and the presence of DNA methylation (meCpG) or H2A.Z are color-coded as indicated. Exact experimental conditions are described in (Lukauskas et al, 2024). The bottom panels display the SNAP SILAC Heavy/Light ratios (Ratio H/L) binding data for human SAGA or ATAC complex subunits as heatmap visualizations obtained from the Modification Atlas of Chromation States (https://marcs.helmholtz-munich.de/). The Heavy/Light ratios for each subunit in each SNAP experiment are shown on a logarithmic (log2) scale as circles, with the right half representing the log2[Ratio H/L] of the forward and the left half the reverse experiment. Binding (H/L ratios >1) and exclusion (H/L ratios <1) are indicated in red and blue colors, respectively. Missing data points where no protein was detected were left blank. Circle sizes denote the mass spectrometry signal detected for each protein. Only data for human SAGA- or ATAC-specific subunits that were detected with sufficiently high data quality are shown. Specific subunits belonging to either human SAGA or ATAC complexes are clustered by similarity of their binding behavior.

To further investigate the potential similarities and differences in chromatin modification pattern recognition by endogenous human SAGA and/or ATAC complexes, we took advantage of a recently published large-scale nucleosome affinity purification database (Lukauskas et al, 2024). To understand how different chromatin readers recognize different chromatin states, the Bartke lab has implemented an unbiased multidimensional MS-based chromatin profiling strategy combining large-scale nucleosome affinity purification with MS approaches to detect the difference in binding between a modified and the unmodified nucleosomes (Lukauskas et al, 2024). To systematically profile the interactomes of chromatin modifications in a nucleosomal context, SILAC nucleosome affinity purifications (SNAP) were performed (Bartke et al, 2010). Using this dataset and the available bioinformatics tools (Lukauskas et al, 2024), we searched for modified nucleosomes that were bound by the human SAGA and/or ATAC complexes (Fig. 2). During our analyses we noted a series of similarities and differences: (a) neither human SAGA nor ATAC would bind to nucleosomes containing histone H2A.Z, heterochromatic histone modifications (H3K9me2/3, H3K27me2/3 and H4K20me3) or methylated DNA (Fig. 2); (b) both human SAGA and ATAC would moderately bind to H3K4me3-modified nucleosomes as predicted by the presence of SGF29 in both complexes (Bian et al, 2011), (c) human ATAC would strongly bind to either H3K4me3-K9ac double-modified nucleosomes, or to H3K4me3-K9ac-K18ac triple-modified nucleosomes; d) no significant binding of human SAGA to these specific double- or triple-modified nucleosome combinations could be detected (Lukauskas et al, 2024) (Fig. 2). In addition to these differences, both human SAGA and ATAC showed differential binding to several modified nucleosomes harboring of H3K4-methylation together with acetylation of histones H3 and/or H4 (Fig. 2), with some combinations inducing strong binding. However, due to the complexity of the modifications on these nucleosomes and the limited number of nucleosomes carrying modifications suited for dissecting SAGA or ATAC binding further, no additional conclusions could be drawn from this study. Nevertheless, these observations suggest that the co-occurrence of several distinct histone modification-binding/recognition domains either in human SAGA or human ATAC (Table 1) differentially regulates the chromatin reading/recognition capabilities of these complexes.

In agreement with this suggestion, chromatin immuneprecipitation coupled to high-throughput sequencing (ChIP-seq) indicated that human SAGA and ATAC are targeted to different genomic loci representing functionally distinct regulatory elements (Krebs et al, 2011). While human SAGA can principally be found at a specific set of promoters, human ATAC is recruited to a different subset of promoters and also to enhancers. Note that SAGA is able to act in the gene body regions very transiently to remove the ubiquitin group from histone H2Bub1 (see below). Furthermore, the *Drosophila* (d) ATAC complex has been suggested to have a general stimulatory effect on nucleosome remodeling by ATP-dependent remodeling complexes, while alone dATAC did not show nucleosome sliding activity (Suganuma et al, 2008). These findings further indicate important functional differences between SAGA and ATAC co-activator complexes at the level of genome localization and/or organization (Fischer et al, 2021; Krebs et al, 2011; Nagy et al, 2010; Suganuma et al, 2008).

## How are SAGA and ATAC recruited to their target sites?

**Figure 3:**
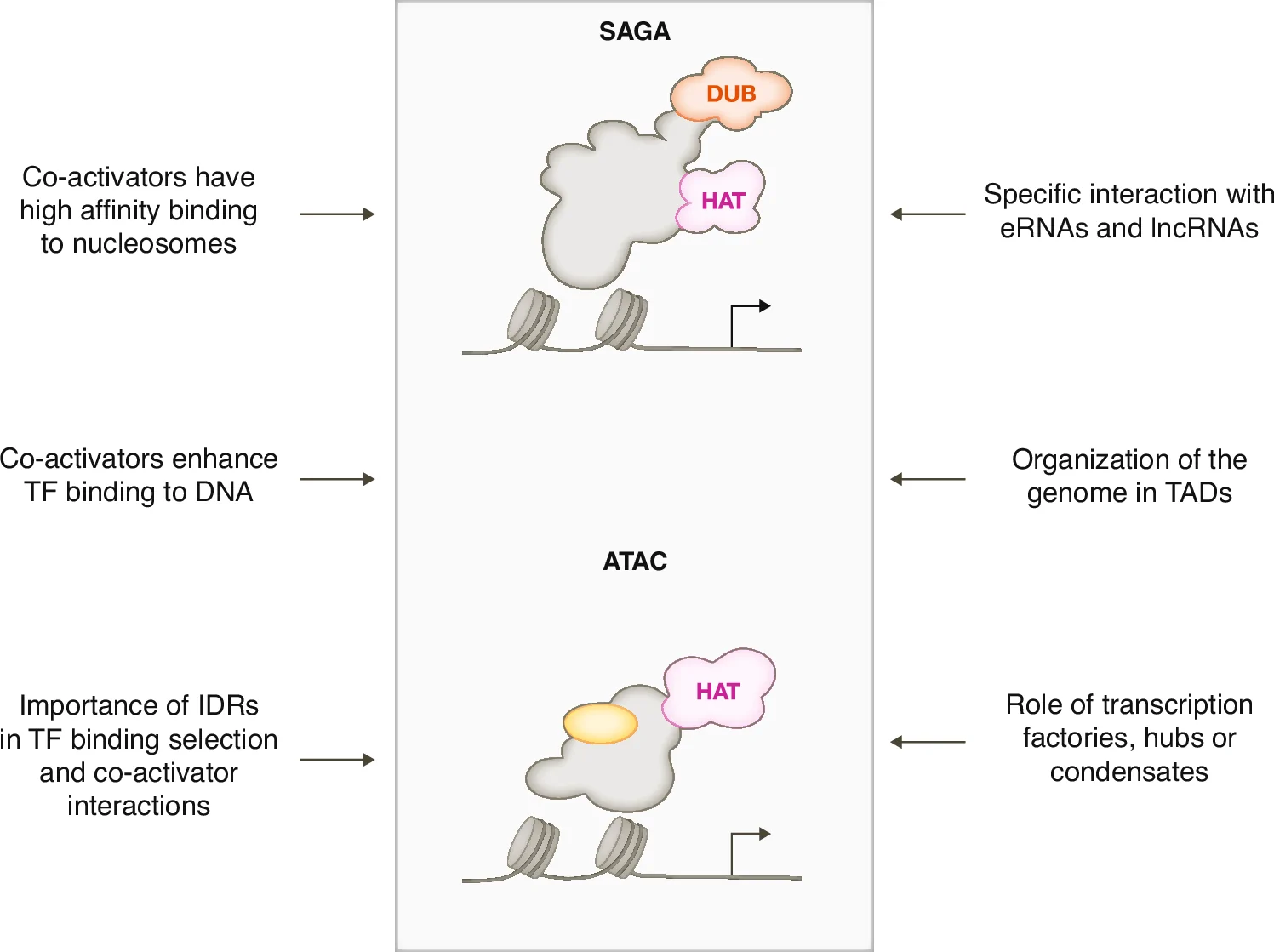
The different layers that can further finetune the action of the SAGA and ATAC co-activators.

**Figure 4:**
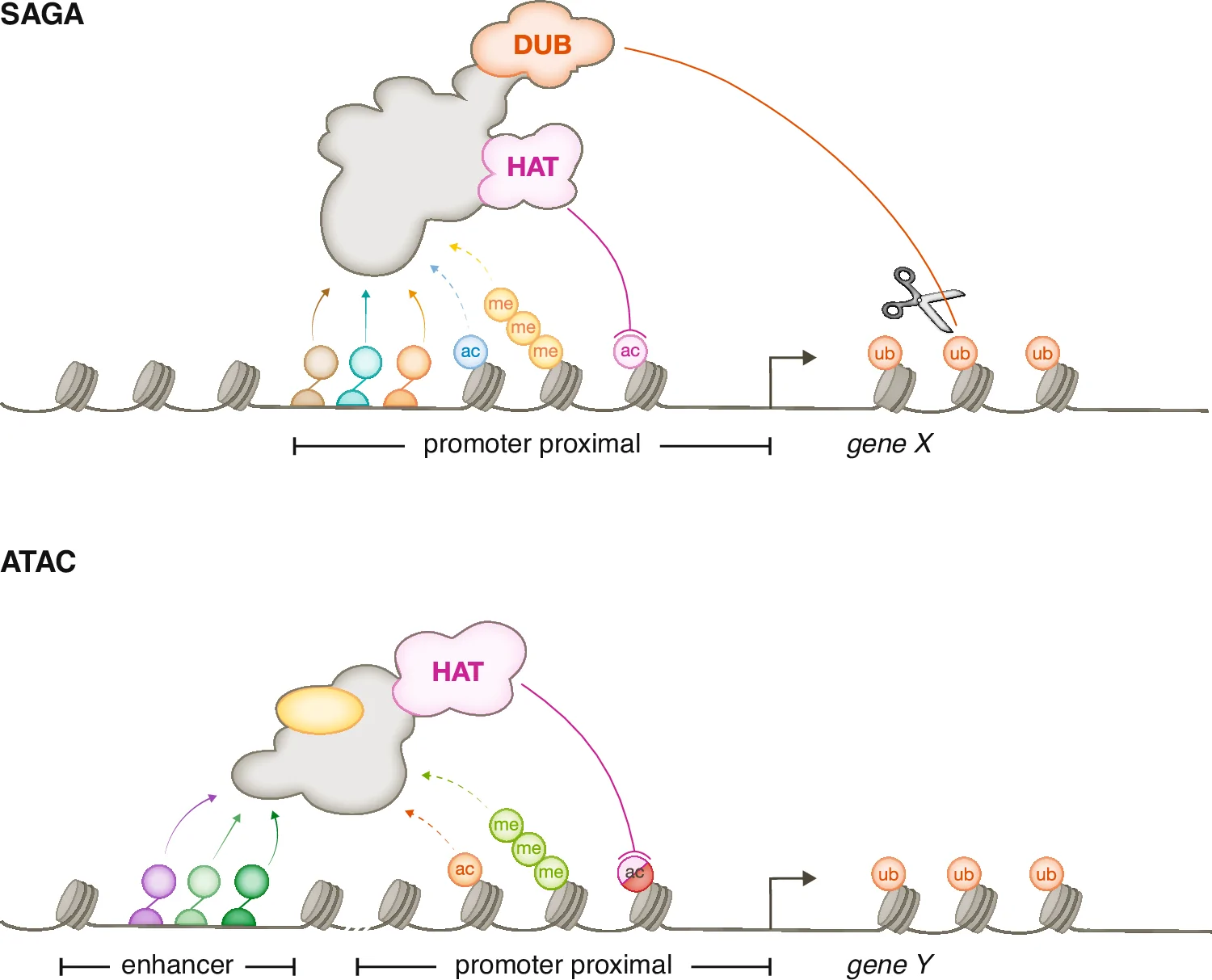
SAGA and ATAC complexes are recruited by distinct activators and chromatin modifications to different regulatory regions to write and erase chromatin modifications and thus regulate distinct set of genes.

How SAGA and ATAC are mechanistically recruited to their target nucleosomes is not fully understood. The commonly-held view is that transcription factors (TFs) that bind their cognate sites within nucleosomal DNA (with affinities in the 2–15 nanomolar range) recruit co-activators, which in turn modify nearby nucleosomes and alter the local chromatin environment (Fig. 3) (Fernandez Garcia et al, 2019). These modifications can also fine-tune the target selection, which will be recognized via multiple “reader” domains. However, this picture ignores several observations: (i) co-activators are already endowed with a high affinity binding (in the 20–50 nanomolar range) to nucleosomes (Mittal et al, 2018) and conceivably to differentially modified nucleosomes (Fig. 2), (ii) vice versa co-activators can also enhance the binding of TFs to DNA (Chesnutt et al, 2024), (iii) TF binding to chromatin is not only dependent on their DNA-binding domains, but also on their intrinsically disordered regions (IDRs), which play an important role in selecting their DNA-binding site (Liu et al, 2026a), iv) co-activators can interact with enhancer RNAs (eRNAs) or long noncoding RNAs (lncRNAs) to stabilize their chromatin binding and/or regulate transcription (Bose et al, 2017; Hemphill et al, 2023; Lai et al, 2013; Long et al, 2017), v) the organization of genomes into topological associated domains (TADs) (da Costa-Nunes and Noordermeer, 2023), vi) the role of higher-order assemblies (i.e., transcription factories, 3D transcriptional hubs or transcriptional condensates) in the regulation of transcription (Navalkar et al, 2026; Papantonis and Cook, 2013) (Fig. 4). Hence, a molecular perspective emerges that includes several synergistic effects and formation of multivalent interaction layers, that together confer an advantage for a given location over other sites for sufficient time to stimulate transcription initiation by Pol II.

## Differential enzymatic activities of SAGA and ATAC complexes

The resembling HAT modules of SAGA and ATAC complexes (Table 1) have been suggested to have similar histone acetylation targets. In agreement, human SAGA and ATAC are known to acetylate H3K9 and H3K14 in a cellular chromatin environment (Bonnet et al, 2014b; Brand et al, 1999; Feller et al, 2015; Fischer et al, 2021; Grant et al, 1999; Nagy et al, 2010; Riss et al, 2015). Along the same lines, when recombinant histone octamers were acetylated in vitro using recombinant SAGA or ATAC HAT modules, acetylation of histone H3, but also histone H4, was observed at very similar lysine positions, namely at H3K9 + K14, H3K14, H3K23, H3K18 + K23, H3K27 + K36, H3K27 + K36 + K37, H3K79 + K91, and H4K5 + K8, H4K8, H4K8 + K12 (Riss et al, 2015). These observations indicate that SAGA and ATAC may carry out similar chromatin “writing” activities.

Interestingly, the ATAC core was described to harbor a second potential acetyltransferase (AT) as its ATAC2 subunit (also called KAT14, Cysteine-rich protein 2-binding protein or CRP2BP) containing a HAT domain related to the GNAT family (Helmlinger et al, 2021). In *Drosophila* cells, it was shown that the ATAC complex possesses different substrate specificity than SAGA, since ATAC acetylates mainly histone H4 (Ciurciu et al, 2006; Guelman et al, 2006; Suganuma et al, 2008). In *Drosophila*, the histone H4K16-specific acetylation activity was suggested to result from the presence of ATAC2/KAT14 in the dATAC complex (Suganuma et al, 2008). However, in a systematic search for non-redundant specificity of HATs (or KATs) in *Drosophila* KC cells, the RNAi knockdown (KD) of ATAC2/KAT14 did not result in reductions of any acetylation at individual lysine residues using mass spectrometry as a read-out, suggesting that other HATs may have redundant or overlapping acetylation functions with ATAC2/KAT14 (Feller et al, 2015). Knock-out (KO) of the *Atac2* gene in mice resulted in the reduction of H3K9ac, H4K5ac, H4K12ac, and H4K16ac modifications, further suggesting that ATAC2/KAT14 may have acetylation specificity towards histone H4 (Guelman et al, 2009). It is conceivable that the KO or KD of *Atac2* is not only ablating the expression of ATAC2 in the cells tested, but may also influence the assembly of the whole ATAC complex and as a consequence change the combined “double” HAT activity of the whole ATAC complex. Thus, the histone acetylation specificity of the entire ATAC complex harboring two potential HATs (KAT2A or KAT2B and ATAC2) remains to be further investigated. Nevertheless, the above observations suggest that *Drosophila* and mammalian SAGA and ATAC complexes have different HAT specificities. Importantly, these chromatin-modifying activities have been described to occur at the promoters of the SAGA- or ATAC-regulated genes (Fischer et al, 2021). However, both human SAGA and ATAC seem to have also distinct non-histone targets when their acetyltransferase (AT) activities were interrogated in detail (Fournier et al, 2016; Yayli et al, 2023). It was shown that the entire human SAGA complex assembles in the cytoplasm, before import to the nucleus and thus, SAGA can acetylate both cytoplasmic and nuclear proteins. In contrast, the fully assembled human ATAC complex can only be detected in the nucleus, where it can acetylate histones and additional protein targets (Fournier et al, 2016; Yayli et al, 2023). Surprisingly, the HAT activity of either SAGA or ATAC was not required for mouse ESCs' survival or to regulate transcription of different target genes in these cells (Fischer et al, 2021).

SAGA has a second chromatin-modifying activity, as it harbors deubiquitylation (DUB) activity towards monoubiquitinated (ub1) histone H2B (H2Bub1) (Daniel et al, 2004; El-Saafin et al, 2022; Henry et al, 2003; Zhang et al, 2008; Zhao et al, 2008). Such DUB activity or any enzymatic activities other than HAT (or AT) are absent from ATAC. The DUB module of the SAGA complex is composed of the ubiquitin-specific protease 22 (USP22) and three adapter proteins (ATXN7/L1/L2, ATXN7L3, and ENY2), which are needed for the full activity of the USP22 enzyme (Lang et al, 2011; Morgan and Wolberger, 2017). Establishment of the H2Bub1 mark by histone ubiquitinase RNF20/40 is directly coupled to active transcription by Pol II and is promoted by several transcription elongation factors, such as for example the polymerase-associated factor complex 1 (PAF1), and the histone chaperone complex, facilitates chromatin transcription (FACT) (Fleming et al, 2008; Pavri et al, 2006; Zhu et al, 2005). As the RNF20/RNF40 complex is traveling with Pol II during transcription elongation, H2Bub1 is deposited exclusively on the gene bodies and not at the promoters of actively transcribed genes (Briggs et al, 2002; Pavri et al, 2006). Indeed, ChIP-seq revealed that H2Bub1 is found at gene bodies of expressed genes and is absent from non-expressed chromosomal regions (Bonnet et al, 2014a; Bonnet et al, 2014b; Chandrasekharan et al, 2010; Fuchs et al, 2014; Pavri et al, 2006; Wang et al, 2021). Thus, repeated cycles of histone H2B ubiquitination by RNF20/RNF40 traveling with transcribing Pol II and SAGA-dependent deubiquitylation of H2Bub1 occur in gene bodies of actively transcribed genes (Briggs et al, 2002; El-Saafin et al, 2022; Kim et al, 2013; Lee et al, 2007). Note, however, that the removal of H2Bub1 by SAGA is not fully understood, as in mammals, related non-SAGA DUB modules could also be responsible for this action (Atanassov et al, 2016; Wang et al, 2021). The SAGA DUB activity has also been implicated in the deubiquitylation of several other cellular proteins, such as subunits of Mediator complex (MED16 and MED24), a multi-subunit transcriptional co-activator, the largest subunit of Pol II, RPB1, the endoplasmic reticulum (ER) chaperone HSPA5 (also known as BIP or GRP78), and receptor-interacting protein kinase 3 (RIPK3) (McCann et al, 2020; Prokakis et al, 2021; Roedig et al, 2021; Stanek et al, 2021). Moreover, the genome-wide SAGA DUB activity on H2Bub1 seems to participate in recycling ubiquitin to constitute a rather limiting nuclear pool that is important during cellular homeostasis and embryonic development (Bonnet et al, 2014b; El-Saafin et al, 2022; Wang et al, 2021).

In conclusion, the SAGA complex “writes and erases” chromatin at distinct two gene regions: around specific promoter regions, where it acetylates histones, and in gene body regions, where it removes H2Bub1 (Bonnet et al, 2014b; El-Saafin et al, 2022). For promoter recruitment of SAGA, DNA-specific TFs and a given chromatin environment seem to be necessary. Note, however, that it is not well understood at present how SAGA is recognizing H2Bub1 deposited in the gene body regions of all transcribed genes, where its genome-wide deubiquitylation action is extremely efficient, when transcription elongation is arrested (Bonnet et al, 2014b; El-Saafin et al, 2022). In contrast, ATAC has been suggested to be recruited to enhancer and promoter regions by DNA-specific TFs and a given chromatin environment to regulate genes, which are different from those controlled by SAGA (Fischer et al, 2021; Krebs et al, 2011) (Fig. 3).

## Differential functional requirements of SAGA or ATAC for normal cellular homeostasis and/or cancer progression

To better understand the role of SAGA and ATAC in mammalian cells, a comprehensive and comparative analysis of these two complexes was carried out in mESCs. CRISPR-Cas9-mediated gene knock-out (KO) technology was used to target genes coding for subunits specific to either the SAGA or ATAC complexes, as well as HAT subunits shared between SAGA and ATAC (Fischer et al, 2021). KO of genes coding for SAGA-specific subunits, such as *Supt7* and *Supt20h* or others, resulted in viable mESC colonies (Fischer et al, 2021; Pijnappel et al, 2013). Surprisingly, KO of genes encoding subunits of ATAC, such as *Yeats2* or *Zzz3*, is supposedly lethal in mESCs as no colonies could be recovered without expression of *Yeats2* or *Zzz3* (Fischer et al, 2021). Similarly, no human ESC colonies could be recovered when the *ZZZ3* gene was knocked out (Lo Conte et al, 2024). When *Supt7* or *Supt20h* (coding for SAGA subunits) were knocked out, or the expression of *Yeats2* or *Zzz3* (coding for ATAC subunits) was knocked down, the loss of these SAGA or ATAC subunits severely affected mESC growth and self-renewal. Furthermore, these experiments showed that these two complexes regulate different subsets of genes (Fischer et al, 2021). Inactivation of SAGA decreased expression of genes related to LIF signaling and self-renewal, whereas ATAC depletion mainly affected the expression of genes related to cytoplasmic translation, such as ribosomal protein coding genes (RPG) (Fischer et al, 2021). The regulation of ribosome biogenesis, translation, and mTOR signaling by down-regulation of several subunits of the ATAC complex has also been reported (Lo Conte et al, 2024; Mi et al, 2017). Thus, it seems that each complex makes use of its specific activities to regulate different sets of genes, which is in agreement with studies carried out in *Drosophila* or in human cancer cell lines (Arede et al, 2022; Gamper et al, 2009; Krebs et al, 2011; Nagy et al, 2010; Pankotai et al, 2005; Pankotai et al, 2010).

Interestingly, a series of unbiased genome-wide CRISPR/Cas9 KO screens identified genes coding for specific SAGA or for common SAGA/ATAC subunits as being crucial for diverse cellular functions in many distinct cellular systems (Doring et al, 2017; Goh et al, 2021; Lo et al, 2025; Shalem et al, 2014; Shankar et al, 2026; Soares et al, 2021; Tzelepis et al, 2016). A genome-wide in vivo hematopoietic stem and progenitor cell (HSPC)-based CRISPR KO screen identified gene hits, including well-characterized hematopoiesis regulators, such as RUNX1 and GATA3, but also genes coding for SAGA complex subunits, *Tada2b, Tada1, Taf5l*, and *Taf6l*. Loss of these SAGA subunits led to the accumulation of immature HSPCs in the bone marrow and a loss of mature blood cells in the peripheral blood following transplantation. At the transcriptional level, this was associated with an upregulation in interferon (IFN) response genes in HSPCs ex vivo and in vivo, likely due to the increased secretion of IFN(α/β). This was also associated with reduced mitochondrial activity and an increased megakaryocyte progenitor cell commitment. In summary, this study identified the SAGA complex as an important regulator of hematopoiesis (Shankar et al, 2026). In the context of this, SAGA KD studies further suggested that it exerts control of cell identity by fine-tuning normal progression of erythroid programs. This role for SAGA may be contributory rather than absolute, as was also suggested by the interaction between GATA1 and TAF10 (Arede et al, 2022; Papadopoulos et al, 2015).

Acute myeloid leukemia (AML) is a heterogeneous disease of the hematopoietic system arising from the accumulation of mutations in hematopoietic stem and progenitor cells. AML is a devastating disease in need of new therapies to improve patient survival. CRISPR drop-out screen in AML cell lines captured several SAGA subunit coding genes (*KAT2A, SUPT20H, TRRAP, TAF5L, TAF6L, TAF10*, and *TAF12*) as AML-specific cell-essential genes potentially targetable for genetic vulnerabilities (Tzelepis et al, 2016). Another targeted CRISPR/Cas9 screen identified genes that cause susceptibility of AML cells to double-negative T cell (DNT) therapy. This screen indicated that inactivation of the subunits of the SAGA DUB module sensitized AML cells to DNT-mediated cytotoxicity (Soares et al, 2021). The mechanistic understanding of SAGA function during normal hematopoiesis and in leukemic states may help to develop SAGA-specific therapies.

In a CRISPR/Cas9 screen that was set up to identify candidate MYCN-amplified neuroblastoma dependency genes as compared to other non-neuroblastoma solid tumor cell lines, genes encoding SAGA subunits like *TADA2B, TADA1, SUPT20H,* and *TAF5L* scored high (Malone et al, 2024). Thus, the SAGA complex was shown to maintain the oncogenic transcriptional state in AML, which suggests a therapeutic potential for inhibitors of SAGA activity in MYCN-amplified neuroblastoma (see below).

Genome-wide CRISPR/Cas9 KO screens were also used in A375 melanoma cells. These cells harbor a gain-of-function *BRAF-V600E* mutation, and they are resistant to vemurafenib, a therapeutic drug inhibiting the mutant BRAF protein kinase. Importantly, several independent CRISPR/Cas9 KO screens identified a series of genes coding for human SAGA subunits, such as *TADA2B*, *TADA1, TAF5L, TAF6L, SUPT20H, TADA3*, and *SGF29 (CCDC101)* for their involvement in establishing vemurafenib resistance (Goh et al, 2021; le Sage et al, 2017; Shalem et al, 2014). How the SAGA complex is able to promote vemurafenib resistance in melanoma cells is not yet understood. Regardless, these studies suggest that a better understanding of SAGA-regulated process(es) will generate new therapeutic strategies for countering melanoma. Most of these studies identified subunits of the HAT and core modules of SAGA. However, these screens rarely identified any DUB module subunits, which are already associated with cancer. Indeed, USP22 is part of the death-from-cancer signature (Glinsky, 2005). Overexpression of USP22 represents a bad prognosis for patients (Glinsky et al, 2005), and fusion of *ATXN7L3* gene (coding for another DUB module subunit) to the *UBTF* gene has been identified in a subclass of B-acute lymphoblastic leukemia (Kimura et al, 2022; Passet et al, 2022).

In contrast, no ATAC-specific subunits were identified in the above-cited screens (Arede et al, 2022; Doring et al, 2017; Goh et al, 2021; Lo et al, 2025; Malone et al, 2024; Shalem et al, 2014; Shankar et al, 2026; Soares et al, 2021; Tzelepis et al, 2016). The fact that these CRISPR/Cas9 KO screens did not allow the identification of viable cell lines lacking specific ATAC subunits suggests that KOs of genes coding for ATAC subunits are lethal, similarly as described in mouse or human ESCs (see above). Alternatively, it is also possible that ATAC has not been thoroughly investigated in other contexts.

## SAGA and ATAC function in distinct DNA repair processes

Understanding how genes influence drug responses is critical for advancing personalized cancer treatments. Large-scale CRISPR-based genetic screens combined with single-cell approaches were applied in primary human 3D gastric organoids to systematically identify genes that affect sensitivity to cisplatin, which is a DNA-damaging chemotherapeutic drug. Surprisingly, *TAF6L* was identified in this screen as a regulator of cell recovery factor from cisplatin-induced cytotoxicity (Lo et al, 2025). As TAF6L is a core subunit of SAGA (Table 1), it seems that the entire SAGA complex influences the cellular viability and proliferation following cisplatin treatment in gastric organoids. In addition, a double-stranded break repair profiling screen upon perturbation of more than 18,000 human genes identified the involvement of the SAGA subunits (TADA1, TADA2B, TAF5L, TAF6L, SUPT20H, SGF29) in microhomology-mediated end joining (de Alba et al, 2025). In this respect, it is important to note that several previous studies have also described connections between SAGA function and DNA repair in human cells (Brand et al, 2001; Evangelista et al, 2018; Martinez et al, 2001). Furthermore, it was found that stabilization of the mismatch repair protein MSH6 in response to alkylation damage requires interactions with the molybdopterin synthase associating complex (MPTAC) and the ATAC complex, and that MSH6 promotes sterol biosynthesis via a MPTAC- and ATAC-dependent manner (Suganuma and Workman, 2022). However, the precise molecular mechanisms underlying the role of SAGA or ATAC in DNA repair remain to be elucidated. From a clinical perspective, SAGA and ATAC may offer therapeutic strategies to explore and evaluate novel pharmacological interventions for treatments in diverse tumor types.

## ATAC may function through regulating translation and/or RNA binding

Beyond its role in interactions with TFs, recognizing modified chromatin patterns, and functioning as a HAT or KAT, several lines of evidence suggest that subunits of the ATAC core complex may be involved in regulating translation and/or the binding of distinct classes of RNA molecules. It has been demonstrated that the translation initiation of iron-responsive mRNA is facilitated by the association of molybdopterin synthase with the human ATAC complex and the subsequent inhibition of a double-stranded RNA-dependent protein kinase (Suganuma et al, 2016). As it has been described that human ATAC functions mostly in the nucleus (Yayli et al, 2023), it is conceivable that ATAC has an indirect effect on translation in the cytoplasm.

Noncoding RNAs produced from enhancers and promoters, including lncRNAs and eRNAs, have been proposed to influence gene expression by binding to transcriptional co-activators and chromatin regulators (Hemphill et al, 2023; Lai et al, 2013; Long et al, 2017). A systematic search for TFs that could bind RNA, regardless of whether they have a known RNA-binding domain or not, found that a substantial fraction of TFs also bind RNA in mESCs and several human cell lines. A combined analysis of data from this and multiple other studies identified 286 RNA-binding proteins with high confidence (Oksuz et al, 2023). Interestingly, the ATAC-specific core subunit, ZZZ3, was identified among these RNA-binding proteins, and it was suggested that its MYB-HTH/SANT domain may be involved in RNA binding (Oksuz et al, 2023). In agreement, a high-throughput study that set out to identify in-cell RNA-protein interactions identified ZZZ3 as an interactor of the *Xist* lncRNA, involved in mammalian X-chromosome inactivation (Graindorge et al, 2019). While the functional relevance of *Xist* binding to ZZZ3/ATAC complex is not yet understood, the identification of ZZZ3 as a lncRNA interactor further suggests that the whole ATAC complex can bind regulatory RNAs.

YEATS2, another ATAC-specific core subunit, has been shown to interact with eRNAs, which play critical roles in gene regulation (Roy et al, 2025). In pancreatic ductal adenocarcinoma (PDAC), activation of a cancer-specific super-enhancer drives production of a MYC-associated eRNA, whose levels correlate with increased *MYC* transcription in patient samples. Moreover, Roy and colleagues demonstrated that the eRNA–YEATS2 interaction is modulated by inflammatory signaling, which promotes recruitment of the ATAC complex to MYC regulatory regions and results in elevated MYC expression (Roy et al, 2025). Consistently, YEATS2 KD reduces *MYC* mRNA levels, underscoring its functional role in MYC transcriptional activation. These findings are in good agreement with the notion that YEATS2 functions as an oncogenic driver, and with its reported overexpression in non-small cell lung cancer (Mi et al, 2017) and hepatocellular carcinoma (Du et al, 2024). Collectively, these data suggest that disrupting YEATS2–eRNA interactions may represent a promising therapeutic strategy.

These findings together support a broader model in which ATAC components may contribute to gene regulation via RNA translation or RNA-mediated mechanisms, potentially coordinating context-specific transcriptional responses in both physiological and pathological settings. It is thus conceivable that eRNAs and certain lncRNAs can act as molecular tethers to couple transcriptional output with chromatin acetylation.

## Small-molecule inhibitor-linked therapeutic strategies for counteracting SAGA and/or ATAC targeting chromatin during cancer cell development

BDs have emerged as therapeutically attractive targets for a number of disease areas, including inflammation and cancer development (Muller et al, 2011; Vidler et al, 2012). Multiple small-molecule inhibitors have been developed against BD and extra-terminal domain (BET) family proteins with promising therapeutic efficacy (Gajjela and Zhou, 2023; Zhang et al, 2026). At their C-termini, both KAT2A and KAT2B contain highly homologous BDs (Fig. 1 and Table 1, and see above) (Li and Shogren-Knaak, 2009; Nagy and Tora, 2007). Nevertheless, the exact function of the KAT2A and KAT2B BDs in mammalian cells is not yet well understood. In 2011, GlaxoSmithKline (GSK) generated the GSK4027 chemical probe for the KAT2A/KAT2B bromodomain, which is selective, potent, and cell penetrable (Humphreys et al, 2017; Moustakim et al, 2017). Numerous small-molecule inhibitors of BD proteins have been further developed into heterobifunctional protein degraders known as proteolysis-targeting chimeras (PROTACs) (Gadd et al, 2017; Lai and Crews, 2017). GSK4027 was developed into the PROTAC GSK699-1 or GSK983, which was shown to degrade both KAT2A and KAT2B in several human cell types, to reduce H3K9ac and to modulate inflammatory signals in macrophages and dendritic cells, and to reduce neuroblastoma viability (Bassi et al, 2018; Malone et al, 2024). These experiments revealed the therapeutic potential of these KAT2A/KAT2B PROTACs. Moreover, GSK4027 when conjugated to desthiobiotin and coupled to streptavidin beads has also proven to be efficient in a novel affinity-ligand purification method to obtain SAGA and ATAC complexes from two different unmodified cell lines (Damilot et al, 2026).

Recently, a chemical probe was developed to inhibit SGF29 recruitment to H3K4me2/3-modified chromatin called SGF29-IN-1 by interfering with the function of its chromatin-reading Tudor domains (Chan et al, 2024), and it would be worthwhile to develop this compound into a PROTAC.

Chromatin and transcriptional dysregulation are defining features of diffuse midline glioma (DMG). Pharmacologic inhibition of shared HAT module subunits of the SAGA and ATAC complexes, using GSK4027 to inhibit KAT2A/KAT2B or SGF29-IN-1 to block SGF29 chromatin binding, suppressed DMG growth relative to control cell lines. These findings suggest that pharmacological interference with SAGA and ATAC activity represents a potential strategy for cancer treatment (Richard et al, 2026).

To achieve greater specificity, recent efforts have shifted toward selective inhibition of the ATAC-specific subunit YEATS2 rather than the shared HAT module. YEATS2, a noncatalytic component of ATAC, recognizes histone H3K27 acylation marks through its YEATS domain (Fig. 1 and Table 1) (Mi et al, 2017). A rationally optimized tetrapeptide inhibitor, LS-170, binds the YEATS domain of YEATS2, reduces ATAC chromatin occupancy at defined loci, and represses ATAC-dependent transcriptional programs, resulting in marked suppression of tumor growth in a lung cancer mouse model (Liu et al, 2026b). These results highlight selective inhibition of the ATAC/YEATS2 axis as a more precise therapeutic approach compared with strategies directed at shared HAT subunits.

Table 1 summarizes known protein domains within SAGA and ATAC subunits, several of which may provide tractable interfaces for small-molecule intervention or PROTAC-based degradation strategies. Although small-molecule inhibitors of SAGA and/or ATAC used alone or in combination therapies have not yet been optimized for clinical application, these advances provide a strong rationale for the development of targeted strategies in contexts where suppression of SAGA- and/or ATAC-dependent functions is therapeutically desirable.

## Conclusions and perspectives

This review integrates the latest functional and molecular insights to provide a comprehensive view of the functions of the related SAGA and ATAC co-activator complexes relevant for the regulation of chromatin functions. We have delineated the interplay amongst DNA-bound TFs, the epigenetic landscape, and the writing/erasing activities of SAGA and ATAC complexes relevant for a proper regulation of transcription in different model systems. We have also summarized recent results that highlight distinct functional requirements of SAGA and ATAC for the maintenance of cellular homeostasis and for oncogenic transformation. Together, this indicates that dysregulation of SAGA and ATAC functions contributes to cancer progression. We also provide an overview of efforts to develop small-molecule inhibitors targeting key components of SAGA and/or ATAC.

Looking ahead, several questions stand out for future research. First, further mechanistic insight is required to understand the exact molecular actions of the SAGA and ATAC co-activator complexes at the single gene level and in different cellular systems. Secondly, targeted PPI measurements under different cellular conditions will uncover the dynamic interactions steering transcription initiation by Pol II. Thirdly, using endogenous or recombinant complexes and chromatinized templates applying in vitro single-molecule biophysics with label-free and fluorescence imaging will be equally important to measure binding forces of SAGA and ATAC and their interactions with TFs on different modified nucleosome-containing templates. Fourthly, super-resolution imaging analyses in living cells will allow decoding the dynamic functions of SAGA and ATAC in vivo. Fifthly, more attention should be given to the non-chromatin-related actions of SAGA and ATAC, which are still poorly explored. Finally, the development of efficient and specific cell-penetrable small-molecule inhibitors offers promising strategies to control chromatin recruitment of SAGA and/or ATAC and their transcriptional activity in cancer cells. Collectively, our review dissects the unique and overlapping functions of SAGA and ATAC, which are crucial for understanding transcriptional regulation in order to exploit these complexes as targets in precision oncology.

## Supplementary information

Peer Review File
