# Supplementary material for: Step-brothers in arms, SAGA and ATAC co-activator complexes, use different strategies
Source: EMBO J. 2026 Aug 11;45(19):6682–98. doi: 10.1038/s44318-026-00892-z (PMC13631227; doi:10.1038/s44318-026-00892-z)
Supplement: Supplementary file 1 [file 44318_2026_892_MOESM1_ESM.pdf]

THE EMBO JOURNAL

Step-Brothers in arms, SAGA and ATAC co-activator complexes, use different strategies

Francesca Rizzo, Adam Ben-Shem, H.Th.Marc Timmers, Till Bartke, Stephane Vincent, and Laszlo Tora

Corresponding author(s): Laszlo Tora (laszlo@igbmc.fr)

Review Timeline:

Submission Date:	23rd Mar 26
Editorial Decision:	23rd Apr 26
Revision Received:	8th Jun 26
Editorial Decision:	15th Jul 26
Revision Received:	17th Jul 26
Accepted:	24th Jul 26

Editor: Cornelius Schneider

Transaction Report:

(Note: With the exception of the correction of typographical or spelling errors that could be a source of ambiguity, letters and reports are not edited. Depending on transfer agreements, referee reports obtained elsewhere may or may not be included in this compilation. Referee reports are anonymous unless the Referee chooses to sign their reports.)

Dear Dr. Tora,

Thank you for submitting your manuscript for consideration by the EMBO Journal. It has now been seen by three referees whose comments are shown below.

Given the referees' positive recommendations, I would like to invite you to submit a revised version of the manuscript, addressing the comments of all three reviewers. I should add that it is EMBO Journal policy to allow only a single round of revision, and acceptance of your manuscript will therefore depend on the completeness of your responses in this revised version.

When preparing your letter of response to the referees' comments, please bear in mind that this will form part of the Review Process File, and will therefore be available online to the community. For more details on our Transparent Editorial Process, please review our Editorial Policies: https://link.springer.com/partners/embo-press/editorial-policies

We generally allow three months as standard revision time. As a matter of policy, competing manuscripts published during this period will not negatively impact on our assessment of the conceptual advance presented by your study. However, we request that you contact the editor as soon as possible upon publication of any related work, to discuss how to proceed. Should you foresee a problem in meeting this three-month deadline, please let us know in advance and we may be able to grant an extension.

Thank you for the opportunity to consider your work for publication. I look forward to your revision.

Yours sincerely,

Cornelius Schneider, PhD
Editor
The EMBO Journal
c.schneider@embojournal.org

Instructions for preparing your revised manuscript:

Read our guidance for manuscript revisions and related editorial policies: https://link.springer.com/journal/44318/submission-guidelines#cms-Revised-submissions

Please make sure you upload a letter of response to the referees' comments together with the revised manuscript.

Please also check that the title and abstract of the manuscript are brief, yet explicit, even to non-specialists.

When assembling figures, please refer to our figure preparation guideline in order to ensure proper formatting and readability in print as well as on screen:
https://media.springernature.com/original/springer-cms/rest/v1/content/27825798/data/v1

At EMBO Press we ask authors to provide source data for the main manuscript figures. You will receive a separate email with instructions for providing source data with your revised manuscript, including how to upload and organize the files.

IMPORTANT: When you send the revision we will require
- a point-by-point response to the referees' comments, with a detailed description of the changes made (as a word file).
- a word file of the manuscript text.
- individual production quality figure files (one file per figure)
- a complete author checklist
- Expanded View files (replacing Supplementary Information)
- a Reagents and Tools Table as part of the Methods section

Please remember: Digital image enhancement is acceptable practice, as long as it accurately represents the original data and conforms to community standards. If a figure has been subjected to significant electronic manipulation, this must be noted in the figure legend or in the 'Materials and Methods' section. The editors reserve the right to request original versions of figures and the original images that were used to assemble the figure.

We realize that it is difficult to revise to a specific deadline. In the interest of protecting the conceptual advance provided by the work, we recommend a revision within 3 months (22nd Jul 2026). Please discuss the revision progress ahead of this time with the editor if you require more time to complete the revisions.

Referee #1:

This is a solid and comprehensive review describing the mechanisms involved in gene regulation by the SAGA and ATAC histone acetyltransferase complexes. The review focuses on the mammalian complexes and covers some of the new studies that have been published since one of the last major reviews focusing only on SAGA was published in 2020 (PMID:33004486). The only minor comment I have is to specify in the figure and table legends which organism (i.e., human) the protein names represent. This would be helpful for more general readers because there are some differences in subunit names for these complexes between species.

Referee #2:

Overall
The regulation of super enhancers has emerged as a focus in the chromatin and transcription fields. In light of these and other studies, the roles of transcription co-activators, nucleosome acetylation, and bromodomain function are of renewed and increasing interest. Hence, reviewing SAGA and ATAC is of interest for not only chromatin researchers but also those studying different areas of genome function.

In this review, the authors introduce the SAGA and ATAC complexes by describing their functional subunit modules. Moreover, the introduction of domains in each subunit are very useful for understanding this manuscript and understanding SAGA and ATAC activities. However, the review is not very comprehensive and focuses a large part on speculation about recent preliminary data while omitting other more detailed mechanistic biochemical studies. Besides obvious differences in subunit composition, most of the discussed differences in SAGA and ATAC functions (the topic of the review) is based on screening data. However, screening results generally need to be proven by more vigorous approaches. In this era of big data, speculating on the data from large screens is not unusual, however, as such this article is more of a "Perspective" about potential differences in SAGA and ATAC functions rather than a "Review" what is known in the field.

Major comments
1. Introduction: There are other publications showing rigorous purifications of SAGA and ATAC from different species. The authors should cite more of these publications to introduce these complexes. 2. Fig.1. Hcf in Drosophila and HCFC1 in humans are bona fide subunits of ATAC (Nature Structural & Molecular Biology 15, 364-372 (2028); JBC 283, Issue (49)2008, Pages 33808-33815). In addition, MAP3K7 is in human ATAC (JBC 283, Issue (49)2008, Pages 33808-33815). The authors did not include these factors in the model illustration shown in Fig. 1. However, the authors should at least mention these subunits in the text.
3. Page 5. Lines 7-14. The authors may compare SAGA in different species with interacting transcription factors in each species. In addition, the authors should clarify which species are being referred to in comments throughout the manuscript.
4. Page 5. Lines 18-19 "Differences in SAGA.... ". It is difficult to understand what authors hope to explain through this sentence.
5. Page 7. Lines 13-21. Page 9 Line 11~. The authors simply concluded that SAGA simply association with TFs in transcription regulation. There additional mechanisms contribute to transcription. ATAC has been shown to facilitate nucleosome remodeling (Nature Structural & Molecular Biology 15, 364-372 (2028)). Thus, the authors have missed this additional potential mechanism of ATAC function in the transcription regulation.
6. Page 8, Lines 5-8. Page 17-18 (section "small molecule"). "Chromatin binding domains" should be replaced with "histone binding domains". Additionally, the authors should briefly describe functions of bromodomains and Tudor domains within SAGA binding modified histones.
7. Page 8, Lines 19-25. This may make confusion to the audience as it may be seen as a contradicted sentence. The authors should describe "combination of" H3K4me3-H3K9ac if it is what the authors hoped to explain.
8. Page 8, Line 26. The conclusion for SAGA nucleosome binding through this screening assay is too careless. The authors should comment with citation of EMBO J 30 (14): page 2829-2842 (2011) that Sgf29 binds histone H3K4me2/3 and is required for SAGA complex recruitment and histone H3 acetylation. Also, the authors should clarify whether SAGA does not prefer PTMs in nucleosome binding, or SAGA really does not bind nucleosomes. For the latter case, the authors should qualify the SAGA ChIP-seq comments described in page 9 as ChIP-seq for non-nucleosome binders must by definition be indirect. In addition, discussion of nucleosome binding through the screening data shown in Fig. 3 is less rigorous.
9. Page 9, Section "How are SAGA and ATAC" as well as Figs 4 and 5 should be in a section labeled "perspective".
10. Page 13, Lines21-26. The authors need to explain the methods. It is difficult to understand the screening system through the current description. What does "build-up" mean?

11. Page 14, Lines 24-27. Not only SAGA subunits but also ATAC subunits.
12. Page 15. ATAC functions in MMR. The authors should mention with a citation (Redox Biology 51: 102270:2022).
13. Page 16, section of ATAC-RNA binding. Besides RNA binding activity, ATAC regulates translation initiation. This mechanism has been clarified (Journal of Molecular Cell Biology 8 (1): Pages 44-50:2016). Since ATAC-RNA binding in current manuscript is meaningless, citing the previous study with a slightly different angle of RNA contact may make this section interesting.
14. With regard to the Bio-ID screen. The authors mention several reasons for potential "false negatives" (transcription factors not found to interact) in this screen. There are also reasons why "false positives" could come out of the screen. Hence it is prudent for the authors to qualify presentation and discussion of this data as it is not as rigorous as other direct biochemical approaches. For example, their table (figure 2) could indicate whether each interaction was detected by Bio-ID screen, biochemical assays, crosslinking, etc. perhaps by using *,#, etc with descriptions in the legend.

Referee #3:

Rizzo et al. present a detailed and well-curated review of the current research surrounding two related coactivation complexes, SAGA and ATAC. This work effectively covers the key components of each complex's activities, comparing and contrasting individual protein subunits as well as the genetic, biochemical, and cellular pathways through which they function. The authors also analyze and elaborate on publicly available data to strengthen their ideas and discussion. Additionally, they address the clinical significance of each complex and relevant pharmacological advances in the context of associated disease states. Finally, they conclude with important future directions that will be important for further elucidating the functions of both complexes in normal and disease contexts.

Taken together, this review fills a notable gap in the literature, as few sources are as current or comprehensive in the field of SAGA and ATAC research. Aside from some minor suggestions, I believe this review will be a valuable addition to the existing literature. Below are several comments for consideration to enhance and improve the piece.

1) On page 5 it was suggested that SAGA is required for all pol II genes, but this comment was made without a citation. However, it should be noted that Frank Pugh came to a somewhat different conclusion (PMID: 36302553).

2) The review may benefit from brief discussion of HAT module activity independent of core complex

3) There is an assumption that SAGA's DUB activity is working in gene bodies in addition to promoters. But in yeast, there is also UBP10, which is thought to operate more in gene bodies. It would be helpful to acknowledge the removal of H2Bub1 by SAGA is not fully understood and perhaps suggest alternate DUBs in mammals could also be responsible for this action.

4) Page 9: argument may benefit from contrasting binding affinity of nucleosomes (included) with that of TF's (not included)

5) Page 10: reviewing the enzymatic activity of SAGA and ATAC, highlight the sites that are modified in yeast. Helps the argument that what's seen in-vitro from SAGA/ATAC could be physiologically significant and just missed in-vivo.

6) Page 14: transition between hematopoiesis and AML is abrupt

7) Page 15 : mention that ATAC has not been thoroughly investigated in other contexts (not shown to be significant in AML or hematopoiesis, but clarify it just hasn't been looked at in detail in other places)

8) Page 17: why KAT2A and WDR5 "potentially" present together in ATAC? Does the research suggest these interactions could be independent of ATAC?

Referee #1:

This is a solid and comprehensive review describing the mechanisms involved in gene regulation by the SAGA and ATAC histone acetyltransferase complexes. The review focuses on the mammalian complexes and covers some of the new studies that have been published since one of the last major reviews focusing only on SAGA was published in 2020 (PMID:33004486). The only minor comment I have is to specify in the figure and table legends which organism (i.e., human) the protein names represent. This would be helpful for more general readers because there are some differences in subunit names for these complexes between species.

We thank the Reviewer for her/his positive comments.
As requested, we have carefully indicated throughout the revised manuscript, the figure and table legends which organism the protein names represent. In addition, we indicated in Figure 1 that the protein names correspond to the human subunit names.

Referee #2:

The regulation of super enhancers has emerged as a focus in the chromatin and transcription fields. In light of these and other studies, the roles of transcription co-activators, nucleosome acetylation, and bromodomain function are of renewed and increasing interest. Hence, reviewing SAGA and ATAC is of interest for not only chromatin researchers but also those studying different areas of genome function.

We thank the Reviewer for her/his positive comments and her/his encouragements.

Major comments

1. Introduction: There are other publications showing rigorous purifications of SAGA and ATAC from different species. The authors should cite more of these publications to introduce these complexes.

As requested, we have included more references to introduce these complexes identified from different species.

2. Fig.1. Hcf in Drosophila and HCFC1 in humans are bona fide subunits of ATAC (Nature Structural & Molecular Biology 15, 364-372 (2028); JBC 283, Issue (49)2008, Pages 33808-33815). In addition, MAP3K7 is in human ATAC (JBC 283, Issue (49)2008, Pages 33808-33815). The authors did not include these factors in the model illustration shown in Fig. 1. However, the authors should at least mention these subunits in the text.

As requested, this point has been added to the legend of Figure 1.

3. Page 5. Lines 7-14. The authors may compare SAGA in different species with interacting transcription factors in each species. In addition, the authors should clarify which species are being referred to in comments throughout the manuscript.

In the revised version, as requested, we have made efforts to better represent this point in revised Table 2 (originally Figure 2). To answer Reviewer 1's and 2's query we carefully indicated which species are being referred to the text, the figure and table legends.

4. Page 5. Lines 18-19 "Differences in SAGA.... ". It is difficult to understand what authors hope to explain through this sentence.

The thank the Reviewer pointing out this discrepancy, we have changed the sentence.

5. Page 7. Lines 13-21. Page 9 Line 11~. The authors simply concluded that SAGA simply association with TFs in transcription regulation. There additional mechanisms contribute to transcription. ATAC has been shown to facilitate nucleosome remodeling (Nature Structural & Molecular Biology 15, 364-372 (2028)). Thus, the authors have missed this additional potential mechanism of ATAC function in the transcription regulation.

We apologize for missing this important ATAC function, and we thank the reviewer for pointing this out. This ATAC function has now been mentioned on page 10 (last but one sentence in the revised version).

6. Page 8, Lines 5-8. Page 17-18 (section "small molecule"). "Chromatin binding domains" should be replaced with "histone binding domains". Additionally, the authors should briefly describe functions of bromodomains and Tudor domains within SAGA binding modified histones.

As requested, "chromatin binding domains" have been replaced by everywhere in the text "histone binding domains". The fact that the BD of KAT2A can interact with acetyl lysines and that the double Tudor domain of SGF29 can bind/read H3K4me2/3 modified nucleosomes has been described in the original version on page 17 and 18, respectively. As requested by the Reviewer, we have now moved these two sentences of the revised final version to page 9 (first paragraph in the revised final version), to introduce which subunits of SAGA or ATAC are supposed to bind which modification and we cited the corresponding papers.

7. Page 8, Lines 19-25. This may make confusion to the audience as it may be seen as a contradicted sentence. The authors should describe "combination of" H3K4me3-H3K9ac if it is what the authors hoped to explain.

We modified the sentence to make it clearer.

8. Page 8, Line 26. The conclusion for SAGA nucleosome binding through this screening assay is too careless. The authors should comment with citation of EMBO J 30 (14): page 2829-2842 (2011) that Sgf29 binds histone H3K4me2/3 and is required for SAGA complex recruitment and histone H3 acetylation. Also, the authors should clarify whether SAGA does not prefer PTMs in nucleosome binding, or SAGA really does not bind nucleosomes. For the latter case, the authors should qualify the SAGA ChIP-seq comments described in page 9 as ChIP-seq for non-nucleosome binders must by definition be indirect. In addition, discussion of nucleosome binding through the screening data shown in Fig. 3 is less rigorous.

We apologize if our description of our screening for modified nucleosomes, which were bound by human SAGA and/or ATAC complexes, seemed to be "careless". We have now made efforts to describe our analyses more clearly. We have also cited the EMBO J 30 (14): page 2829-2842 (2011) paper. We are very sorry, if we gave the impression that SAGA does not bind PTMs. Actually, the study by Lukauskas *et al* (2024) detects the difference in binding between a modified and the unmodified nucleosomes. As shown by Figure 2 (originally Figure 3), human SAGA is clearly binding to many modified nucleosomes. We have entirely rewritten this section and the corresponding figure legend to describe clearly our analyses and the resulting findings (see page 9 second paragraph to page 10 first paragraph in the revised version.

9. Page 9, Section "How are SAGA and ATAC" as well as Figs 4 and 5 should be in a section labeled "perspective".

We have included the word "perspective" in the "How are SAGA and ATAC" section.

10. Page 13, Lines21-26. The authors need to explain the methods. It is difficult to

understand the screening system through the current description. What does "build-up" mean?

In this study (PMID: 41577693), the authors perform a genome-wide *in vivo* hematopoietic stem and progenitor cell (HSPC)-based CRISPR knockout screen. The authors discover SAGA complex subunits as key regulators of hematopoiesis We corrected the description. “Build-up” was replaced by “accumulation” in the revised final version.

11. Page 14, Lines 24-27. Not only SAGA subunits but also ATAC subunits.

As in this section we have cited three papers (PMID: 24336571, 33604667, 29255251) we do not know, to which paper is the Reviewer actually referring to. We checked again the corresponding papers and we found only SAGA specific subunits in these three publications.

12. Page 15. ATAC functions in MMR. The authors should mention with a citation (Redox Biology 51: 102270:2022).

We thank the Reviewer for pointing out the role of ATAC in MMR. This ATAC function with the corresponding citation has been included on page 18 (last sentence in the revised final version) and the title of the section has been changed accordingly.

13. Page 16, section of ATAC-RNA binding. Besides RNA binding activity, ATAC regulates translation initiation. This mechanism has been clarified (Journal of Molecular Cell Biology 8 (1): Pages 44-50:2016). Since ATAC-RNA binding in current manuscript is meaningless, citing the previous study with a slightly different angle of RNA contact may make this section interesting.

We are sorry if the Reviewer thought that the ATAC-RNA binding section was “meaningless” in spite of the fact that we cited a few high-profile publications describing this possibility. However, we have now followed the Reviewer’s suggestion and mentioned that ATAC regulates translation initiation and deleted a few sentences from this section to make the section simpler (page 19 second section in the final revised version).

14. With regard to the Bio-ID screen. The authors mention several reasons for potential "false negatives" (transcription factors not found to interact) in this screen. There are also reasons why "false positives" could come out of the screen. Hence it is prudent for the authors to qualify presentation and discussion of this data as it is not as rigorous as other direct biochemical approaches. For example, their table (figure 2) could indicate whether each interaction was detected by Bio-ID screen, biochemical assays, crosslinking, etc. perhaps by using *,#, etc with descriptions in the legend.

We thank the Reviewer for this comment. We have represented the requested information together with the corresponding citations in revised Table 2 (originally Figure 2).

Referee #3:

Taken together, this review fills a notable gap in the literature, as few sources are as current or comprehensive in the field of SAGA and ATAC research. Aside from some minor suggestions, I believe this review will be a valuable addition to the existing literature.

We thank the Reviewer for her/his very positive comments. We were very happy to learn that s/he thought that our review "will be a valuable addition to the existing literature".

1) On page 5 it was suggested that SAGA is required for all pol II genes, but this comment was made without a citation. However, it should be noted that Frank Pugh came to a somewhat different conclusion (PMID: 36302553).

The section has been rewritten and a sentence has been added to emphasize the findings of the Pugh lab (page 6 first paragraph in the revised version).

2) The review may benefit from brief discussion of HAT module activity independent of core complex

As suggested, the findings that the SAGA HAT or DUB modules exist without the core SAGA complex is now mentioned in the Introduction section (see page 4 second paragraph in the revised version).

3) There is an assumption that SAGA's DUB activity is working in gene bodies in addition to promoters. But in yeast, there is also UBP10, which is thought to operate more in gene bodies. It would be helpful to acknowledge the removal of H2Bub1 by SAGA is not fully understood and perhaps suggest alternate DUBs in mammals could also be responsible for this action.

As requested, we have added a sentence on the alternate DUB modules on page 14.

4) Page 9: argument may benefit from contrasting binding affinity of nucleosomes (included) with that of TF's (not included)

As requested, the binding affinity of TFs that bind their cognate binding sites in a nucleosomal context has been added to this section (page 11 second paragraph in the revised final version).

5) Page 10: reviewing the enzymatic activity of SAGA and ATAC, highlight the sites that are modified in yeast. Helps the argument that what's seen in-vitro from SAGA/ATAC could be physiologically significant and just missed in-vivo.

We respectfully do not understand what is the suggestion of the Reviewer as the ATAC complex does not exist in yeast.

6) Page 14: transition between hematopoiesis and AML is abrupt

As requested, a sentence was added to make the transition less abrupt (page 16 third paragraph in the revised version).

7) Page 15 : mention that ATAC has not been thoroughly investigated in other contexts (not shown to be significant in AML or hematopoiesis, but clarify it just hasn't been looked at in detail in other places)

We have rewritten this section and added a sentence as suggested by the Reviewer (page 18 first paragraph in the revised version)

8) Page 17: why KAT2A and WDR5 "potentially" present together in ATAC? Does the research suggest these interactions could be independent of ATAC

We have deleted these two studies to make the RNA-binding section clearer and simpler.

Dear Dr. Tora,

Thank you for submitting a revised version of your manuscript. Your study has now been seen by the original referee #2, who find sthat their previous concerns have been addressed and now recommends publication of the manuscript. There remain only a few mainly editorial points that have to be addressed before I can extend formal acceptance of the manuscript:

- missing funding info: IdEx Unistra (ANR-10-IDEX-0002) and by SFRI-STRAT'US project (ANR 20-SFRI-0012) and EUR IMCBio (ANR-17-EURE-0023); "Funding" should be included in "Acknowledgements"

- Please rename the Conflict of Interest section into "Disclosure and Competing Interests Statement", in accordance with our updated Guide to Authors (https://link.springer.com/partners/embo-press/editorial-policies#Competing%20interest%20disclosures)

- As we are switching from a free-text author contribution statement towards a more formal statement based on Contributor Role Taxonomy (CRediT) terms, please remove the present Author Contribution section and instead specify each author's contribution(s) directly in the Author Information page of our submission system during upload of the final manuscript. See https://casrai.org/credit/ for more information.

- Section order should be corrected: Title page - Abstract - Introduction - Acknowledgements (if any) - Disclosure and Competing Interests Statement - References - Figure Legends - Table(s) (if any)

Please let me know if you have any questions regarding any of these points. You can use the link below to upload the revised files.
Thank you again for giving us the chance to consider your manuscript for The EMBO Journal. I look forward to receiving the final version.
With best regards,
Cornelius Schneider

____
Cornelius Schneider, PhD
Editor | The EMBO Journal
c.schneider@embojournal.org

Please refer to our figure preparation guideline in order to ensure proper formatting and readability in print as well as on screen:

https://link.springer.com/journal/44318/submission-guidelines#cms-Figure-and-data-presentation

----

Referee #2:

The authors have addressed all of my issues in the revised version

The authors have addressed all minor editorial requests.

Dear Dr. Tora,

I am pleased to inform you that your manuscript has been accepted for publication in the EMBO Journal.

Your manuscript will be processed for publication by EMBO Press. It will be copy edited and you will receive page proofs prior to publication. Please note that you will be contacted by Springer Nature Author Services to complete licensing and payment information.

You may qualify for financial assistance for your publication charges - either via a Springer Nature fully open access agreement or an EMBO initiative. Check your eligibility: https://link.springer.com/journal/44318/how-to-publish-with-us

Should you be planning a Press Release on your article, please notify the production team via this form in order to coordinate publication and release dates: https://forms.cloud.microsoft/e/eH4A8JVP6g

If you have any questions, please do not hesitate to contact the Editorial Office. Thank you for your contribution to The EMBO Journal.

Yours sincerely,

Cornelius Schneider, PhD
Editor
The EMBO Journal
c.schneider@embojournal.org

*****
Please note that it is The EMBO Journal policy for the transcript of the editorial process (containing referee reports and your response letters) to be published as an online supplement to each paper. If you should prefer removal of any referee-only figures included in the point-by-point response(s), e.g. because they may still be used for future publication or because they have been reproduced from published work by others, please do let us know immediately via response email.
More information is available here: https://link.springer.com/partners/embo-press/editorial-policies#Peer%20review
